# Modeling Climate‐Driven Vegetation Changes Under Contrasting Temperate and Arid Conditions in the Mediterranean Basin

**DOI:** 10.1002/ece3.70753

**Published:** 2025-01-11

**Authors:** Marco Bianchini, Mohamed Tarhouni, Matteo Francioni, Marco Fiorentini, Chiara Rivosecchi, Jamila Msadek, Abderrazak Tlili, Farah Chouikhi, Marina Allegrezza, Giulio Tesei, Paola Antonia Deligios, Roberto Orsini, Luigi Ledda, Maria Karatassiou, Athanasios Ragkos, Paride D'Ottavio

**Affiliations:** ^1^ Department of Agricultural, Food and Environmental Sciences Università Politecnica delle Marche Ancona Italy; ^2^ Pastoral Ecosystems, Spontaneous Plants and Associated Microorganisms Laboratory Arid Regions Institute‐University of Gabes Medenine Tunisia; ^3^ Department of Civil, Constructional and Environmental Engineering Sapienza University of Rome Rome Italy; ^4^ Laboratory of Rangeland Ecology, School of Forestry and Natural Environment Aristotle University of Thessaloniki Thessaloniki Greece; ^5^ Agricultural Economics Research Institute Hellenic Agricultural Organization – DIMITRA Athens Greece

**Keywords:** climate change, machine learning, pastoral systems, predictive vegetation models, rangelands

## Abstract

This study investigates climate change impacts on spontaneous vegetation, focusing on the Mediterranean basin, a hotspot for climatic changes. Two case study areas, Monti Sibillini (central Italy, temperate) and Sidi Makhlouf (Southern Tunisia, arid), were selected for their contrasting climates and vegetation. Using WorldClim's CMCC‐ESM2 climate model, future vegetation distribution was predicted for 2050 and 2080 under SSP 245 (optimistic) and 585 (pessimistic) scenarios. Two spectral indices, NDVI (temperate area) and SAVI (arid area), served as vegetation proxies, classified into three classes using K‐means (NDVI: high = mainly associated with woodlands, medium = shrublands, continuous grasslands and crops, low = discontinuous grasslands, bare soil, and rocks; SAVI: high = mainly associated with woods, olive trees, and crops, medium = shrublands and sparse olive trees, low = bare soil and saline areas). Classes validated with ESA WorldCover 2020 data and sampled via 1390 presence‐only points. A set of 33 environmental variables (topography, soil, and bioclimatic) was screened using Pearson correlation matrices, and predictive models were built using four algorithms: MaxEnt, Random Forest, XG Boost, and Neural Network. Results revealed increasing temperatures and declining precipitation in both regions, confirming Mediterranean climate trends. Vegetation changes varied by area: in the temperate area, woodlands and shrublands were stable, but discontinuous grasslands expanded. In the arid area, woodlands gained suitable habitat, while bare soil declined under the pessimistic SSP 585 scenario. Both areas showed an upward shift for shrublands and grasslands. The models indicated significant shifts in areal distribution and environmental conditions, affecting habitat suitability and ecosystem dynamics. MaxEnt emerged as the most reliable algorithm for small presence‐only datasets, effectively predicting habitat suitability. The findings highlight significant vegetation redistribution and altered ecosystem dynamics due to climate change, underscoring the importance of these models in planning for future ecological challenges.

## Graphical abstract

This study explores climate change impacts on Mediterranean vegetation in Monti Sibillini (temperate) and Sidi Makhlouf (arid). Using the CMCC‐ESM2 climate model and spectral indices (NDVI, SAVI), predictive models (MaxEnt, Random Forest, XG Boost, Neural Network) showed rising temperatures, reduced precipitation, and vegetation shifts. In temperate areas, discontinuous grasslands expanded; in arid areas, woodlands gained habitat while bare soil declined. Results underline climate‐driven redistribution of vegetation, highlighting MaxEnt's reliability for small datasets and the importance of predictive models for ecological planning.

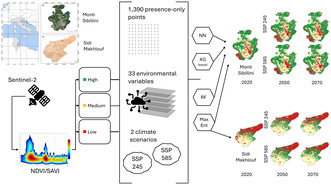

## 1 Introduction

Since the 1980s, climate change has undergone drastic intensification, marked by an increase in temperature extremes, as well as heightened intensity and frequency of weather events such as unpredictable extreme rainfall and droughts. Estimates suggest a growing expansion of arid and semi‐arid areas in the coming decades (Faye et al. 2023; Durán‐Sandoval et al. 2023; Webber et al. 2018). Recent research has highlighted the increasing influence of climate change on the “dry‐wet” polarization, characterized by heightened precipitation in wet areas and exacerbated dryness in arid regions, with tropical and arid zones anticipated to face a growing burden of climate change consequences accordingly (Ouled Belgacem and Louhaichi 2013; Pastor and Khodayar 2023; Scheiter and Higgins 2009; Sun et al. 2022). A common denominator of the climate change is the severe socioeconomic crises it triggers, particularly impacting developing nations. These crises include famine, desertification, disease outbreaks, hydrogeological hazards, mass migration, and wildfires (Durán‐Sandoval et al. 2023; Faye et al. 2023; Webber et al. 2018). Most of the effects of climate change on worldwide agricultural systems include drops in production levels (Webber et al. 2018), scarcity of water (Godde et al. 2020), shifts in crops and fodder phenology (Yang et al. 2023), pest and diseases out brakes (Durán‐Sandoval et al. 2023), and changes in the range of invasive species (Faye et al. 2023).

Mediterranean basin is characterized by low annual precipitation and high interannual variability. It is also reported to be one of the climate change “hot spots” because of changing in ocean's marine heat waves, which are altering Mediterranean climate reducing precipitation, increasing temperatures, and further enhancing variability of extreme events (Pastor and Khodayar 2023; Tuel and Eltahir 2020). These alterations are threatening not only coastlines, freshwater resources, and urban infrastructures but also croplands, grasslands and woodlands (Makris et al. 2023; Pastor and Khodayar 2023). Here, the agricultural sector plays a crucial role in ensuring food security because it serves, as a cornerstone for the well‐being of many communities. Therefore, the adoption of adaptive strategies, such as shifting sowing and harvesting periods, altering crop rotation, and adjusting grazing calendars, have become common practices (Durán‐Sandoval et al. 2023; Faye et al. 2023; Sgroi et al. 2023; Toderi et al. 2017).

Among agricultural systems, pastoral systems are particularly vulnerable to climate change, as they depend on rangelands as a primary forage resource. Climate change is projected to impact livestock mobility and health through reduced rangeland availability, land degradation, conflicts over pastureland access, declines in livestock populations, and decreased availability of water and forage (Durán‐Sandoval et al. 2023; Faye et al. 2023; Godde et al. 2020; Yang et al. 2023). A general reduction in grasslands (with regional variations) is also expected, driven by the encroachment of species well‐adapted to drought and high temperatures (xerophilous), leading to their transformation into shrublands and forests (Godde et al. 2020). These dynamics are confirmed for Italy, as evidenced by Dibari et al. (2015) for the Apennines, and for Africa, where by 2100 significant grassland areas are projected to transition into savannas, with nearly half of the savannas expected to be replaced by deciduous woodlands. This shift is driven by rising atmospheric CO_2_ levels and rainfall patterns that favor woody vegetation over herbaceous species (Scheiter and Higgins 2009; Scholtz et al. 2014). Regarding crops, the impacts of climate change predominantly involve declines in production levels, water availability, shifts in crop phenology, and expansions in the range of pests, diseases, and invasive species. Additionally, significant changes are expected in traditional agricultural practices, including altered sowing and harvesting periods and shifts in cultivation zones (Durán‐Sandoval et al. 2023; Faye et al. 2023).

The adaptation of agricultural and forestry systems to climate change will increasingly require adaptation measures to mitigate its effects. Some of these measures need the involvement of stakeholders at various levels (Eckardt et al. 2023; Godde et al. 2020). Therefore, it is crucial to develop and test predictive models to guide decision‐making, both for conservation efforts and the effective utilization or management of resources. Species distribution and predictive vegetation models are very common methods to forecast the future distribution of single species, communities, and vegetation types. These models employ statistical methods or machine learning algorithms to correlate sample occurrences (presence/absence, or presence‐only) with various environmental variables (usually topographic, soil characteristics, and climatic) as predictors of distribution (Bedair, Shaltout, and Halmy 2023; Elith et al. 2006; Tarkesh and Jetschke 2012). Other very common input variables in predictive models are the spectral indices. Among these, there are the Normalized Difference Vegetation Index (NDVI), which is widely used to assess and classify dense vegetation cover, and the Soil Adjusted Vegetation Index (SAVI), which is more sensitive to sparse vegetation compared to NDVI, as it reduces soil interference (Bannari et al. 1995; Ferchichi et al. 2022; Qu et al. 2024; Wegmann, Leutner, and Dech 2016).

In recent decades, machine learning algorithms have played a pivotal role in predictive vegetation models, evaluating habitat suitability for various vegetation types (Almeida et al. 2023; Beigaitė et al. 2022; Ferchichi et al. 2022; Qu et al. 2024). Notable algorithms in this domain include Maximum Entropy (MaxEnt), Random Forest, eXtreme Gradient Boosting (XG Boost), and Neural Networks. All of these algorithms have been widely applied in different research fields to map fire risks and hydrogeological phenomena (Javidan et al. 2021; Martín, Zúñiga‐Antón, and Rodrigues Mimbrero 2019; Norallahi and Seyed Kaboli 2021), to predict land use/land cover changes (Kavhu, Mashimbye, and Luvuno 2021), also in species distribution models (Blanco, Ameztegui, and Rodríguez 2020; Garzón et al. 2006; Keenan et al. 2011), and predictive vegetation models (Dibari et al. 2015; Ebrahimi et al. 2022; Ferchichi et al. 2022; Qu et al. 2024). Both species distribution and predictive vegetation models produce maps depicting habitat suitability for the spatial distribution of the modeled species or vegetation types (Bedair, Shaltout, and Halmy 2023; Elith et al. 2006; Tarkesh and Jetschke 2012). As such, they might be used as dialogical tools in participatory meetings to facilitate dialog among stakeholders involved in co‐design processes (Toderi et al. 2007, 2017). This would be of great value for complex systems such as pastoral systems providing relevant ecosystem services in the Mediterranean basin (Dean et al. 2024).

The ongoing climate changes in the Mediterranean basin are expected to make arid regions drier and temperate regions wetter, this is likely to impact the distribution of vegetation differently (Sun et al. 2022). Given that the Mediterranean basin encompasses both arid and temperate regions, it is plausible that contrasting Mediterranean climate regions may undergo different vegetational changes. In line with this scenario, four machine learning algorithms in predictive modeling have been utilized, namely MaxEnt, Random Forest, XG Boost, and Neural Network. The first objective of this study was to determine the best‐performing algorithm under the analyzed conditions, which include presence‐only data, small dataset, and varying climate conditions. As a secondary aim, this study seeks to predict vegetation changes based on climate change scenarios in two areas with different vegetation and contrasting climatic characteristics: Monti Sibillini (central Italy, temperate) and Sidi Makhlouf (southern Tunisia, arid).

## 2 Materials and Methods

### 2.1 Study Areas

**FIGURE 1:**
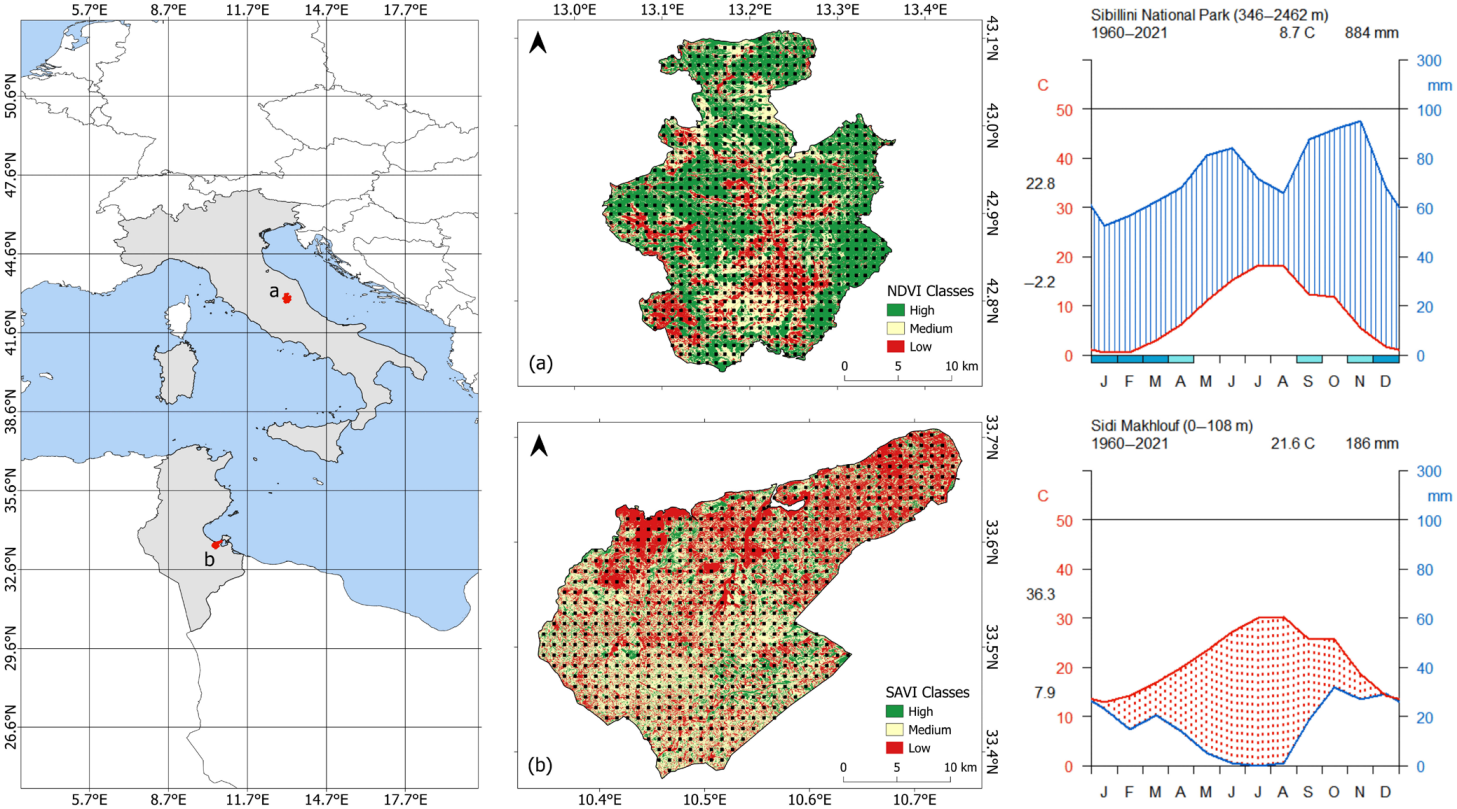
Monti Sibillini (a) and Sidi Makhlouf (b) study areas with their vegetation indices maps classified via K‐means classification (see paragraph 2.2), their occurrence points (black squares), and their Walter‐Lieth diagrams.

Two contrasting Mediterranean areas have been assessed in this study, namely Monti Sibillini National Park (Italy), and Sidi Makhlouf délégation (Tunisia) (Figure 1). Monti Sibillini covers an area of approximately 70,000 ha and is situated in the central sector of the Apennines Mountain range (13.03°E, 42.72°N; 13.37°E, 43.11°N). Sidi Makhlouf covers an area of about 66,000 ha and is in southern Tunisia (10.34°E, 33.37°N; 10.75°E, 33.71°N).

According to the Köppen‐Geiger climate classification (Kottek et al. 2006), the climate in Monti Sibillini is classified as Cfb (warm temperate, fully humid, warm summer), and in Sidi Makhlouf as BWh (arid, desert, hot arid). Worldclim's monthly historical data (Fick and Hijmans 2017) were used to produce Walter‐Lieth climatic diagrams (Figure 1), which confirmed the previous climatic conditions: in the Monti Sibillini temperate climate, precipitations never fall below temperatures and have two peaks in the periods of May–June and September–November. In the Sidi Makhlouf arid conditions, temperatures consistently exceed precipitations, and rainfall reaches its highest values in May and October–November (Figure 1).

Agricultural systems in the Monti Sibillini area rely on traditional pastoral practices with cattle and sheep grazing on grasslands (40% of the area) and some shrublands (Caballero et al. 2009). Croplands focus on winter cereals, grain legumes, lucerne, and sainfoin meadows, while woodlands cover about 50% of the area. In Sidi Makhlouf, pastoral systems are primarily semi‐intensive/extensive, focusing on sheep, goats, and camels grazing year‐round on steppic rangelands (42% of the area). Olive groves, field crops, irrigated orchards, and legumes complement the agricultural landscape (Aribi et al. 2022).

Therefore, from now on, we will refer to the two study areas as the “temperate area” and the “arid area,” respectively.

### 2.2 Vegetation Data

Vegetation indices maps were produced based on the maximum photosynthetic period for each study areas. Sentinel‐2 Level‐2A satellite tiles were downloaded in QGIS (version 3.28.4) via Semi‐Automatic Classification Plugin. For the temperate area, NDVI was calculated on 29 August 2018. For the arid area, SAVI was calculated on 16 April 2018. Here, SAVI was chosen over NDVI because the very sparse vegetation in the study area would exhibit very low NDVI values, potentially introducing bias to the results (Bannari et al. 1995; Ferchichi et al. 2022; Qu et al. 2024; Wegmann, Leutner, and Dech 2016). Vegetation indices were calculated in QGIS, according to the following equations:

(1) $$\text{NDVI}=\frac{\mathrm{NIR}-\mathrm{Red}}{\mathrm{NIR}+\mathrm{Red}}=\frac{8-4}{8+4}$$

(2) $$\text{SAVI}=\frac{\mathrm{NIR}-\mathrm{Red}}{\mathrm{NIR}+\mathrm{Red}+L}\times \left(1+L\right)=\frac{8-4}{8+4+0.5}\times \left(1+0.5\right)$$

where numbers represent the respective Sentinel bands, and *L* in SAVI, is a coefficient ranging from 0 to 1, linked to vegetation cover percentage, and is usually set to the default value of 0.5 following Bannari et al. (1995).

Vegetation maps were then reclassified into “high,” “medium,” and “low” vegetation index classes using a K‐means classification with the SAGA 7.2 tool. To predict NDVI and SAVI changes via distribution models, a regular grid with a mesh size of 0.008333° (≈900 m) was created. This grid was used to sample vegetation index classes, which were then stored in a .csv format file along with coordinates. This process resulted in 763 occurrence points (high: 265; medium: 296; low: 202) for the temperate area, and 627 points (high: 77; medium: 312; low: 238) for the arid area.

The vegetation index classes have been considered as proxies for vegetation types to be modeled. By checking the European Space Agency's global land cover map (Zanaga et al. 2021), it was verified that for the temperate area the High‐NDVI class includes woodlands, the Medium‐NDVI class includes mainly continuous grasslands, the Low‐NDVI class mainly consists of discontinuous grasslands. In the arid area, the High‐SAVI comprises woodlands, olive trees, and crops, the Medium‐SAVI includes shrublands and sparse olive trees, and the Low‐SAVI gathers bare soil and salty areas. For simplicity, where relevant, the main vegetation types associated with the VI classes analyzed in the respective areas will be used in the “Results and discussion” sections.

### 2.3 Environmental Variables

A total of 33 environmental variables (19 bioclimatic, 3 topographic and 11 soil properties) have been considered as predictors for the modelization (Table 1). All the raster variables have been resampled to 0.000269° spatial resolution (≈30 m) with SAGA 7.2, cut to the study area bounds and stored as .asc file in QGIS.

**TABLE 1:** List of the 33 environmental variables considered as predictors for the modelization in Monti Sibillini (MSB) and Sidi Makhlouf (SMK).

Type (Source)	Code	Description	Units	MSB	SMK
Climate (WorldClim), spatial resolution: ≈900 m	bio1	Annual mean temperature	°C	✓	
	bio2	Mean diurnal range (mean of monthly [max temp—min temp])	°C		
	bio3	Isothermality (BIO2/BIO7) (×100)	%		
	bio4	Temperature seasonality (SD × 100)	%		
	bio5	Max temperature of warmest month	°C		
	bio6	Min temperature of coldest month	°C		
	bio7	Temperature annual range (BIO5‐BIO6)	°C		
	bio8	Mean temperature of wettest quarter	°C		
	bio9	Mean temperature of driest quarter	°C		
	bio10	Mean temperature of warmest quarter	°C		✓
	bio11	Mean temperature of coldest quarter	°C		
	bio12	Annual precipitation	mm	✓	✓
	bio13	Precipitation of wettest month	mm		
	bio14	Precipitation of driest month	mm		
	bio15	Precipitation seasonality (coefficient of variation)	%	✓	
	bio16	Precipitation of wettest quarter	mm		
	bio17	Precipitation of driest quarter	mm		✓
	bio18	Precipitation of warmest quarter	mm		
	bio19	Precipitation of coldest quarter	mm		
Topography (Earthdata), spatial resolution: ≈30 m	Elevation	Terrain elevation above the sea level	m		
	Aspect	Slope's orientation from North	°	✓	✓
	Slope	Steepness of the land surface	°	✓	✓
Soil characteristics (SoilGrids 2.0), spatial resolution: ≈250 m	Bulk_D	Bulk density (0–5 cm depth)	cg cm^−3^		
	Coarse_F	Coarse Fragments (0–5 cm depth)	cm^3^ dm^−3^	✓	✓
	Sand	Sand content (0–5 cm depth)	g kg^−1^		
	Silt	Silt content (0–5 cm depth)	g kg^−1^	✓	
	Clay	Clay content (0–5 cm depth)	g kg^−1^		
	CEC	Cation Exchange Capacity at pH 7 (0–5 cm depth)	mmol (c) kg^−1^	✓	✓
	pH	pH in water (0–5 cm depth)	pH 10		
	N	Nitrogen concentration (0–5 cm depth)	cg kg^−1^		
	OCD	Organic carbon density (0–5 cm depth)	hg m^−3^	✓	
	SOC	Soil organic carbon (0–5 cm depth)	dg kg^−1^		
	SOC_Stock	Soil organic carbon stock (0–30 cm depth)	t ha^−1^		✓

Abbreviations: CEC, cation exchange capacity; N, nitrogen; OCD, organic carbon density; SOC, soil organic carbon.

Among the 23 future Global Climate Models developed in the Coupled Model Intercomparison Project Phase 6 program, the CMCC‐ESM2 (Lovato, Peano, and Butenschön 2021) has been selected, as it has proven to be among the best at modeling precipitation and reducing bias in similar areas (Dibari et al. 2020; Gobie et al. 2024; Pimonsree et al. 2023). Two Shared Socio‐economic Pathways (SSP) have been chosen as different scenarios, SSP 245 as optimistic, and SSP 585 as pessimistic trends in greenhouse gasses emissions and downloaded from WorldClim database (Fick and Hijmans 2017) for three time slices (2020, 2050, and 2070).

Pearson's correlation indices were calculated between the predictors to address potential collinearity issues (Table S1). In the matrix, all the variables showing a Pearson's *r* ≥ 0.75 or ≤ −0.75 have been considered highly correlated and therefore discarded, according to Mechergui et al. (2021). However, “Annual mean temperature” and “Annual precipitation” were retained for the temperate area, and “Mean temperature of the warmest quarter” and “Annual precipitation” for the arid area, because they exhibited a high correlation with all the other discarded variables (Table S1). Hence, a total of nine predictors had been retained for the temperate area, and eight predictors for the arid area (Table 1).

### 2.4 Distribution Modeling

Four machine learning algorithms have been employed to predict vegetation indices distribution: MaxEnt, Random Forest, XG Boost, and Neural Network. The MaxEnt version used in this work was 3.4.4. The software was set to disable the auto features option and enable all the available features, Cloglog output format was chosen to carry out a prediction map (from 0 to 1 of presence probability). Basic, Advanced, and Experimental menu options were left as default (Radosavljevic and Anderson 2014). All other machine learning algorithms were executed in R‐Studio version 2023.06.0 Build 421. A 10‐replicated bootstrap run type modelization was carried out for each period and scenario, with a 35% sample test splitting.

The predictive ability of the models was assessed through the “area under the receiver operating characteristics curve” (AUC). The AUC is the probability that a randomly chosen presence cell has a higher predicted value than an absence cell, hence this metric assesses the model's ability to distinguish between locations where the sample is present and locations where it is absent (Elith et al. 2006; Phillips, Anderson, and Schapire 2006). The AUC values range from 0 to 1, with AUC ≥ 0.75 indicating a good performance, 0.5 suggesting a predictive discrimination close to random chance, and values below 0.5 indicating performance worse than random chance (Elith et al. 2006). To assess the contribution of each variable to the predictive model, Jackknife charts were utilized for MaxEnt, while variable importance charts were employed for the other algorithms.

To estimate the areal changes of the three vegetation index classes (i.e., high, medium, and low), the raster layers produced by each algorithm were imported into QGIS and vectorized. In the case of MaxEnt results, it was necessary to merge the presence probability maps for each NDVI class into a single raster. After that, the resulting area and environmental variable average values were calculated for each vegetation indices class and period to be analyzed.

## 3 Results

### 3.1 Current Vegetation, Environmental, and Climate Conditions

In the temperate area, the High‐NDVI class was the most widespread, while the Low‐NDVI class was the least prevalent. High‐NDVI class (woodlands) tended to be predominant at lower elevations compared to Medium‐NDVI (continuous grasslands) and Low‐NDVI (discontinuous grasslands) classes, which extended to higher elevations. It is worth noting that the average elevation for Low‐NDVI is influenced by bare soil in the lowland areas (Table 2). High‐NDVI occupied sites with the highest slopes and south aspect, while Medium‐NDVI and Low‐NDVI classes preferred the least steep slopes and south–south‐west aspect. High‐NDVI class showed to be settled in sites with higher annual mean temperatures, followed by Medium‐NDVI and Low‐NDVI classes, reflecting their elevational distribution (Table 2). In terms of annual precipitation, the High‐NDVI class had the lowest value while the low‐ and medium‐NDVI the highest values. The coefficient of variation of annual precipitation was almost the same for the three vegetation index classes tending to increase from High to Low (Table 2).

**TABLE 2:** Environmental variables (average values) of the Vegetation Indices (VI) classes in the two study areas.

Study area	VI Class	Area (ha)	Elevation (m a.s.l.)	Slope (°)	Aspect (°)	bio1 (°C)	bio10 (°C)	bio12 (mm)	bio15 (%)	bio17 (mm)
Monti Sibillini	High	35,103	1040	23	180	9	—	893	21	
	Medium	22,735	1295	19	192	8	—	916	21	
	Low	12,271	1335	19	190	8	—	914	22	
	Total	70,108	—	—	—	—	—	—	—	—
Sidi Makhlouf	High	8629	36	5	178	—	29	197	—	2
	Medium	32,684	45	6	176	—	29	196	—	2
	Low	24,867	32	5	176	—	29	199	—	2
	Total	66,180	—	—	—	—	—	—	—	—

Abbreviations: bio1 , annual mean temperature; bio10 , mean temperature of warmest quarter; bio12 , annual precipitation; bio15 , precipitation seasonality (coefficient of variation); bio17 , precipitation of driest quarter.

In the arid area, Medium‐SAVI class was the most widespread, followed by Low‐SAVI and High‐SAVI, which occupied the smallest area. Medium‐SAVI class (shrublands and sparse olive trees) tended to occupy the highest elevation, followed by High‐SAVI (woodlands, olive trees, and crops) and Low‐SAVI (bare soil and salty areas) classes (Table 2). Slope was almost the same for the three vegetation index classes changing, on average of 1°C from Medium‐SAVI class, which reached the steepest sites. South was the prevalent aspect in the study area, and it was almost the same for the three vegetation index classes. The mean temperature of the warmest year quarter was slightly higher in Medium‐SAVI class compared to Low‐SAVI. Annual precipitation was highest for Low‐SAVI class, followed by High‐SAVI and Medium‐SAVI classes (Table 2).

### 3.2 Model Performance and Variable Contribution

In the temperate area, MaxEnt, Random Forest, XG Boost, and Neural Network exhibited fair to good performance, with AUCs ranging from 0.67 to 0.71 during the testing phase (Table 3). In the arid area, MaxEnt showed the best performance, followed by Random Forest and XG Boost, and lastly Neural Network. In the arid area, MaxEnt was the only algorithm to achieve a fair prediction AUC (Table 3).

**TABLE 3:** Area under the receiver operating characteristics curve (AUC) values for the training and test samples for each machine learning algorithm and study area.

Model	AUC training		AUC test	
	Monti Sibillini	Sidi Makhlouf	Monti Sibillini	Sidi Makhlouf
MaxEnt	0.83	0.75	0.70	0.62
Random Forest	0.81	0.67	0.70	0.54
XG Boost	0.73	0.53	0.71	0.54
Neural Network	0.65	0.52	0.67	0.53

**FIGURE 2:**
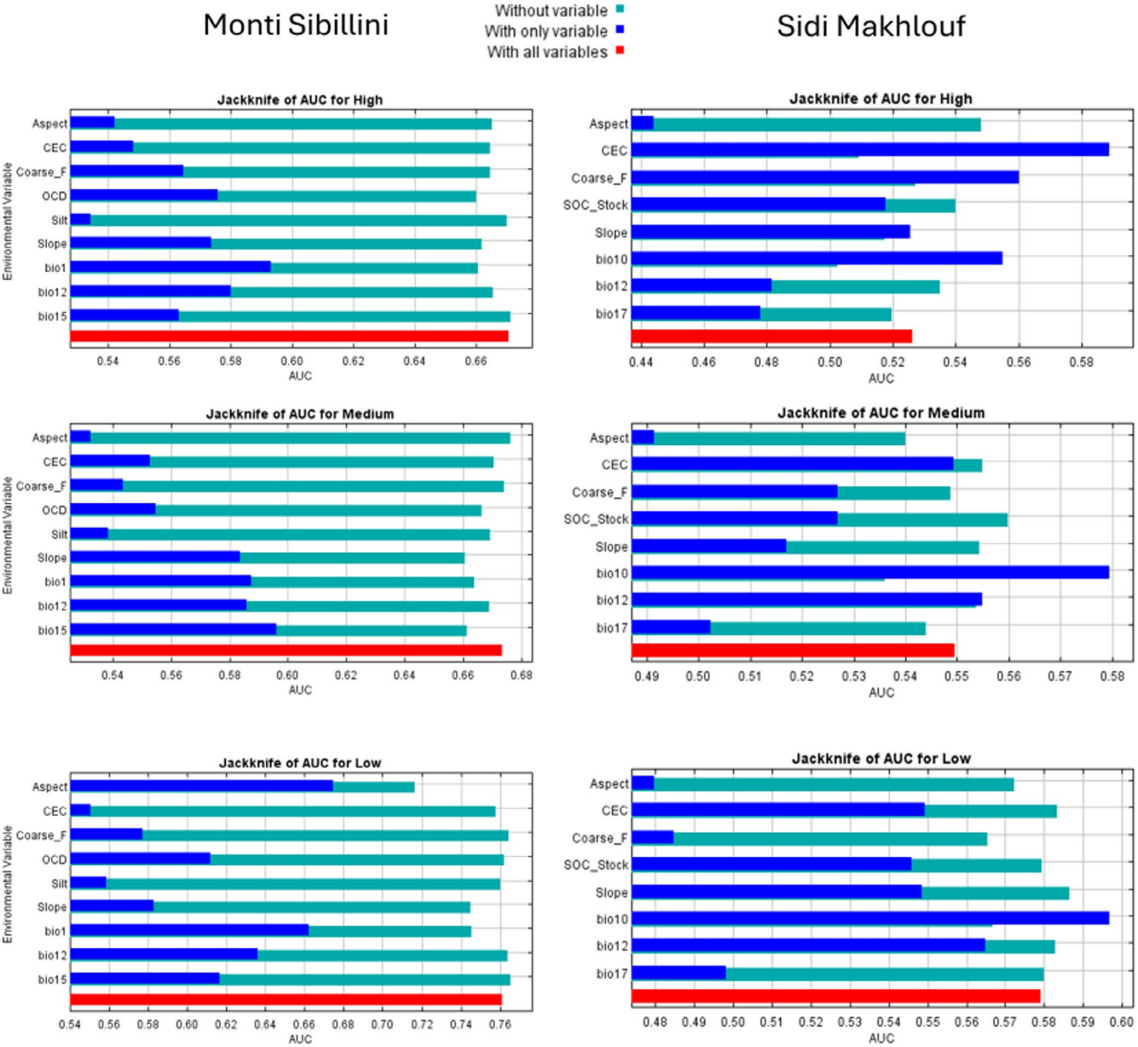
MaxEnt's Jackknife charts for high, medium and low vegetation index classes for the two study areas.

In the temperate area, annual mean temperature and slope were generally the two most contributing variables for almost all the models (Figure 2 and Figure S1). In the MaxEnt jackknife chart for Medium‐NDVI class, precipitation seasonality was most important, whereas for Low‐NDVI aspect was the most important (Figure 2). In the arid area, the two most important variables for almost all the predictive vegetation models were the mean temperature of the warmest quarter and the annual precipitation (Figure 2, Figure S1). The MaxEnt Jackknife chart for High‐SAVI class and Neural Network gave more importance to the cation exchange capacity (Figure 2, Figure S1).

### 3.3 Predicted Vegetation, Environmental, and Climate Conditions

**FIGURE 3:**
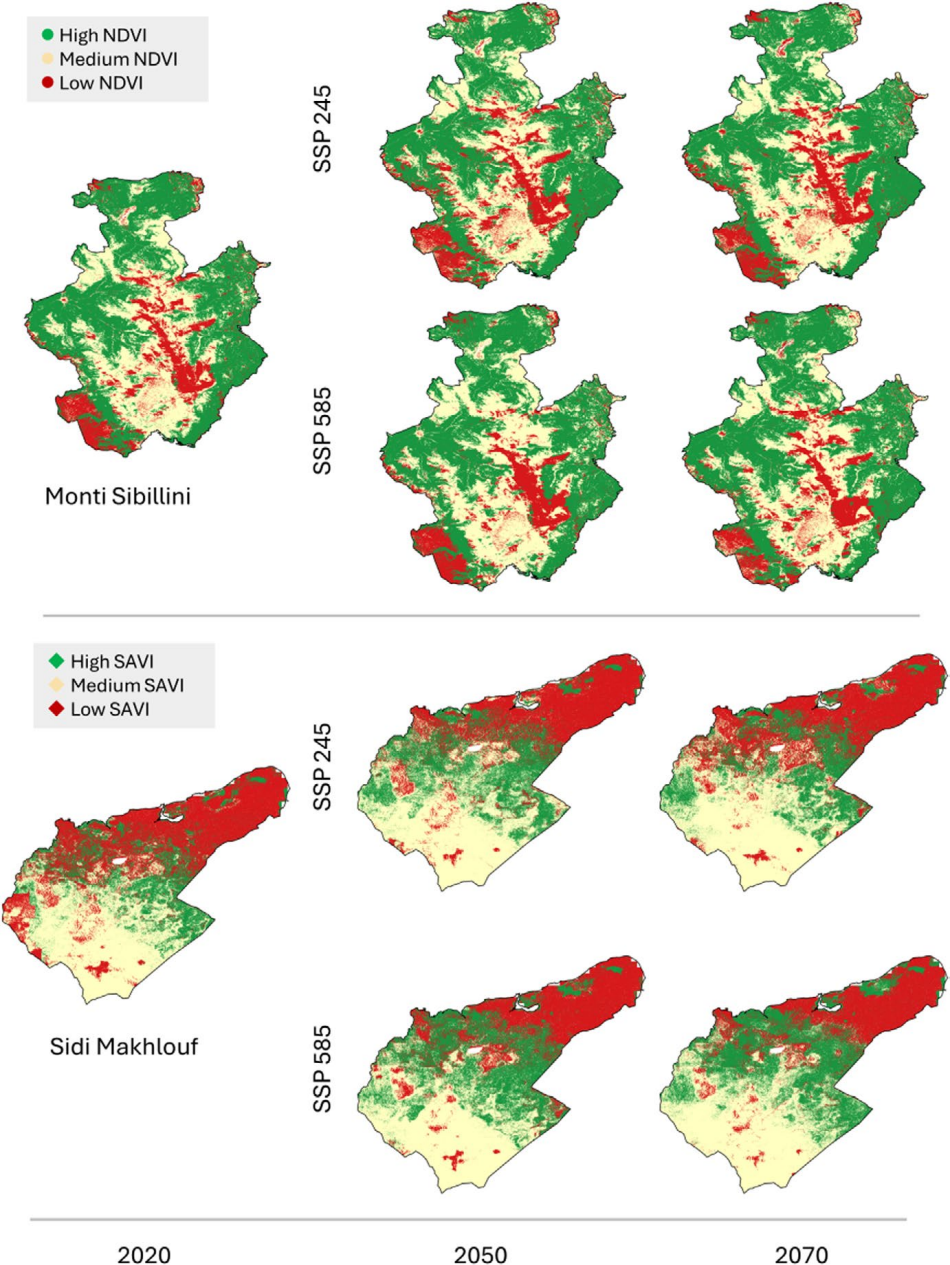
Vegetation changes predicted by MaxEnt for the two study areas across three periods (2020, 2050, and 2070) and two climatic scenarios (SSP 245 and SSP 585). Additional maps predicted by Random Forest, XG Boost, and Neural Network are included in the Supporting Information.

**FIGURE 4:**
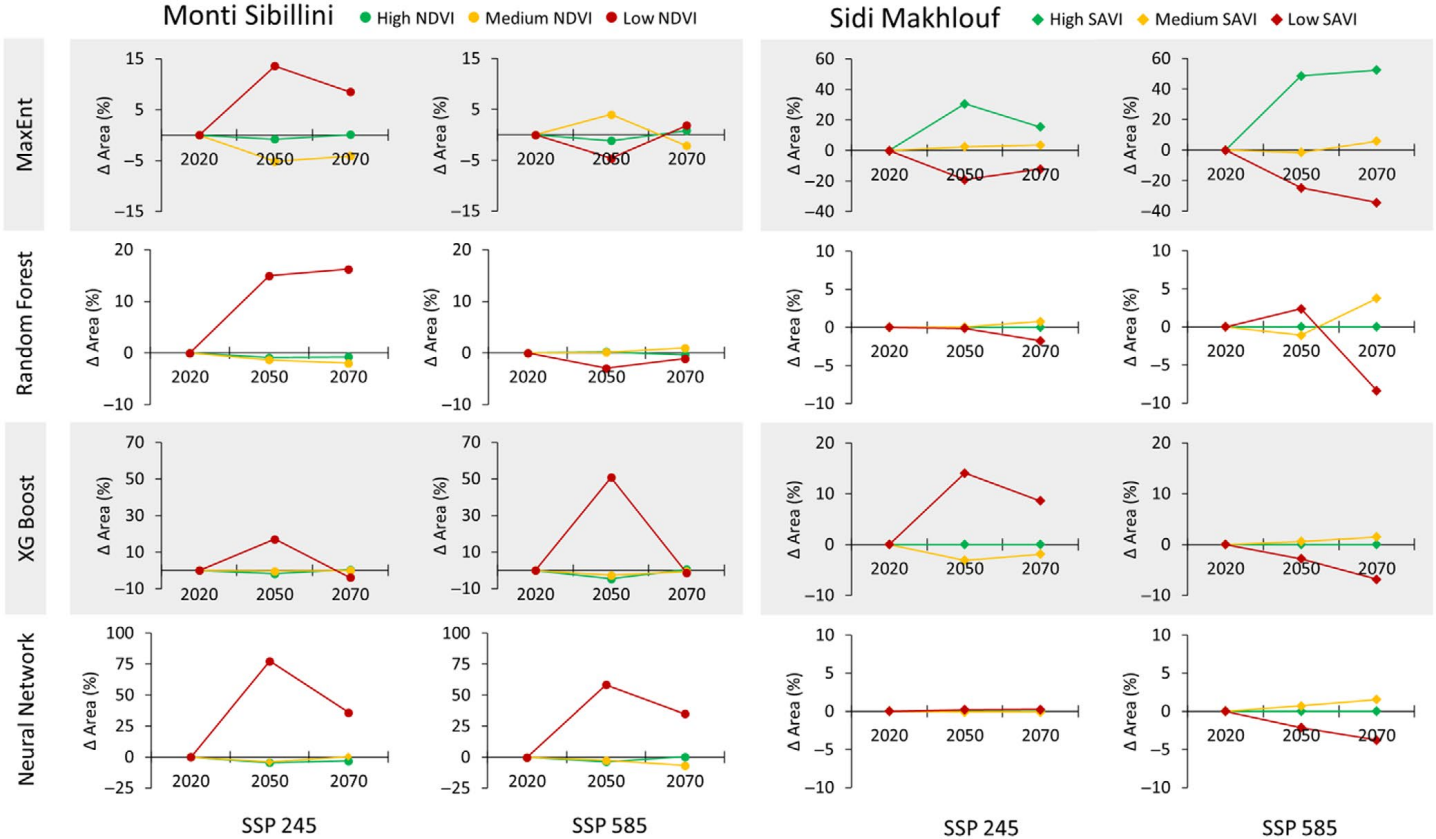
Predicted vegetation change (% change from current conditions) for the two study areas across three periods (2020, 2050, and 2070) and two climatic scenarios (SSP 245 and SSP 585).

In terms of the areal distribution changes, the High‐NDVI (woodlands) and Medium‐NDVI (continuous grasslands) classes in the temperate area remained stable according to nearly all models, irrespective of the “optimistic” SSP 245 or “pessimistic” SSP 585 scenarios. Low‐NDVI class (discontinuous grasslands) was predicted to increase in all the models in the optimistic scenario (on average about +11% from the current condition), except for the Neural Network model which predicted the greatest increase (about +56% on average from the current condition) (Figures 3 and 4; and Figures S2–S4). In the pessimistic scenario, the Low‐NDVI class decreased in the MaxEnt and Random Forest models by an average of −1% and −2%, respectively, compared to the current condition, while it increased in the XG Boost and Neural Network models by approximately +25% and + 47%, respectively (Figures 3 and 4; and Figures S2–S4). In the arid area, MaxEnt was the only algorithm able to model the High‐SAVI (woodlands, olive trees, and crops) class (Table S3), showing a lower increase in the optimistic scenario and a higher increase in the pessimistic one (SSP 585). In the optimistic scenario, the Low‐SAVI class (bare soil and salty areas) exhibited the most significant changes in MaxEnt and XG Boost models, decreasing by about −16% and increasing by approximately +11%, respectively, compared to the current condition, while remaining almost stable in the other models. The Medium‐SAVI class (shrublands and sparse olive trees) remained stable across almost all models, with a slight increase in MaxEnt (approximately +3% on average) and a decrease in XG Boost (about +2% on average) compared to the current condition (Figures 3 and 4; and Figures S2–S4). In the pessimistic scenario, the Low‐SAVI class decreased in all models, with MaxEnt predicting the highest decrease of −30% compared to the other models averaging −4% (Figures 3 and 4; and Figures S2–S4). Conversely, shrublands in the Medium‐SAVI class showed a slight increase in all the models (+1%).

Regarding the analysis of environmental variables, all the models applied in temperate areas were almost consistent in their results in both optimistic and pessimistic scenarios. In this study area, all the models predicted the highest increase in elevation for the Medium‐NDVI class in both optimistic and pessimistic scenarios (on average, 183 m and 191 m, respectively), except for MaxEnt in which this trend was lower (about 75 m on in both scenarios). High‐NDVI and Low‐NDVI, in contrast, decreased in elevation by about −25 m and −45 m, respectively, in the optimistic scenario, and by about −27 m and −48 m in the pessimistic scenario. Low‐NDVI experienced the highest increase in aspect conditions, about 17° and 20° toward southwest in optimistic and pessimistic scenarios, respectively (Table S2). In arid areas, High‐SAVI experienced the most significant shifts in exposure to the west (+34° and +8° in optimistic and pessimistic scenarios, respectively). Medium‐SAVI was the only class to increase in elevation (about +6 m on average in both scenarios) and experienced steeper slopes. Low‐SAVI experienced the greatest decrease in elevation (about −15 m on average in both scenarios) (Table S3).

Regarding the climate conditions, in the temperate area the models generally showed higher bioclimatic changes in the pessimistic scenario compared to the optimistic one (Table S2). All the vegetation index classes will experience increased annual mean temperatures, lower in optimistic and higher in the pessimistic scenario, on average about +3°C and +4°C, respectively, compared to the current conditions. Annual precipitation will decrease for all the vegetation index classes of about −51 mm and −66 mm in the optimistic and pessimistic scenarios, respectively (Table S2). In the arid area, climate conditions are predicted to change mostly with an increased mean temperature of the warmest month (on average +3°C and +4°C in optimistic and pessimistic scenarios, respectively), and a decreased annual precipitation, which is generally consistent across both scenarios. However, the Low‐SAVI class experienced an increase in annual precipitation in the optimistic scenario, 6 mm on average, and a decrease of about −4 mm in the pessimistic scenario (Table S3).

## 4 Discussion

### 4.1 Performance of the Predictive Models Used

Currently, using vegetation indices to predict vegetation changes remains an underexplored method, resulting in limited available studies on the topic. This study contributes to predicting vegetation changes due to climate change in two areas with contrasting climatic characteristics. It employs four different algorithms and two spectral vegetation indices (NDVI and SAVI). Among the few available studies, some combine NDVI with Land Use/Land Cover maps to predict vegetation changes in China using Random Forest, MaxEnt and other algorithms (Qu et al. 2024). Other studies have addressed the prediction of vegetation changes under different climate scenarios using other methods and algorithms, such as Random Forest (Bedair, Shaltout, and Halmy 2023; Keenan et al. 2011), XG Boost and Neural Network (Almeida et al. 2023; Feng et al. 2022; Garzón et al. 2006). However, species distribution and predictive vegetation models may become important tools for stakeholders to cope with the ongoing climate change phenomena. Predictive vegetation models, in particular, can aid in identifying hotspots of environmental change and assessing vegetation habitat suitability. These insights can effectively guide conservation priorities and management planning (Bedair, Shaltout, and Halmy 2023; Javidan et al. 2021; Keenan et al. 2011). Currently, there is still limited literature exploring the prediction of Vegetation Indices as proxies for vegetation distribution, especially with MaxEnt. In this study, MaxEnt has allowed to predict the distribution of a sparsely geographically represented vegetation index class (i.e., High‐SAVI in the arid area) with uniform environmental variables. Indeed, the literature suggests that MaxEnt can be advantageous because it effectively handles small datasets of presence‐only occurrences, which are often the most accessible type of data in certain geographic areas. Moreover, MaxEnt does not require outlier elimination, data transformation, or dataset balancing (Javidan et al. 2021; Mechergui et al. 2021; Noce, Cipriano, and Santini 2023; Qu et al. 2024).

The predictive performance of machine learning algorithms depends, among other factors, on the spatial scale of assessment, spatial resolution of the environmental variables, and dataset size. In this study, a fine resolution of approximately 30 m has been employed, suitable for the sub‐regional scale of assessment. A resolution of 1 km is commonly used in similar analyses to capture larger regional scales and align with the native resolution of the climatic variables (Almeida et al. 2023; Dagnino et al. 2020; Ebrahimi et al. 2022). However, there is also a recognition of the importance of finer‐scale modeling and smaller study areas in ecological research and conservation efforts (Bedair, Shaltout, and Halmy 2023; Blanco, Ameztegui, and Rodríguez 2020; Qu et al. 2024). Nonetheless, fine‐resolution data, such as digital elevation models, are not consistently available or uniformly scaled across different countries. Therefore, the Aster program's digital elevation model employed in this study was considered an appropriate compromise.

Given these conditions, the results of this study were found to be fair‐good in the temperate area, where MaxEnt, Random Forest, and XG Boost achieved nearly identical AUC scores, with Neural network performing slightly worse. The application of multiple machine learning models in an attempt to determine the most suitable for predicting species' habitats has yielded conflicting results. For instance, Feng et al. (2022) reported that XG Boost and Random Forest were the best‐performing models for predicting species' habitats in China. However, XG Boost demonstrated better performance than Random Forest and Neural Network in predicting Land Use/Land Cover in Africa (Kavhu, Mashimbye, and Luvuno 2021). Other studies have instead highlighted that Random Forest outperformed MaxEnt (Qu et al. 2024). In the present study, MaxEnt performances proved to be comparable to the other machine learning algorithms in temperate area. Moreover, in arid area, MaxEnt was the only algorithm able to predict the High‐VI class and gained the highest AUC. Here MaxEnt proved to be the best‐suited algorithm in dealing with small presence‐only dataset (Mechergui et al. 2021) and uniform environmental variables, such as those found in arid and flat areas.

### 4.2 Predicting Vegetation Distribution Changes Under Climate Scenarios

There is increasing evidence suggesting that worldwide vegetation areal distribution will decrease (Almeida et al. 2023; Ben Mariem and Chaieb 2017; Mechergui et al. 2021; Sarikaya and Orucu 2021). In contrast with these findings, all the models applied in temperate areas indicated an increase in Low‐NDVI class (discontinuous grasslands), with no substantial changes in High‐NDVI (woodlands) or Medium‐NDVI (continuous grasslands) classes. The increase in discontinuous grasslands could be a signal that this area is transitioning toward more arid conditions, as already reported elsewhere by Sun et al. (2022), who assessed vegetation patterns as proxy indicators of changing climate conditions. The increase in bare soil could lead to the reduction and fragmentation of priority habitats and a decrease in biodiversity, with serious consequences on the conservation capacity of protected areas (Bedair, Shaltout, and Halmy 2023; Kaky et al. 2020; Mechergui et al. 2021). Dibari et al. (2015) projected a decrease in grassland extension along the Apennines, ranging from 80% to 90% under both optimistic and pessimistic climate scenarios. In their study, the authors utilized a 1 km resolution, while using a 30 m resolution in the current study may have led to minimal changes, as seen here with continuous grasslands. Other authors found slight changes in woodland vegetation. For example, Noce, Cipriano, and Santini (2023) predicted a general reduction of about 16% of woodland areal distribution in the central Apennines. In the present study, all models consistently indicate a slight reduction (−1%) in woodland areas. It is possible that the local woodland vegetation in the temperate area has found its optimum conditions due to the topography, potentially serving as a refuge for woodlands, similarly to what has been reported by Dagnino et al. (2020), who suggested a comparable role for endemic species in the Alps.

In the arid area, all the models showed a decrease in bare soils. Here, only MaxEnt was able to predict the future distribution of woodlands. The increase in this vegetation class suggests that it would benefit from more suitable conditions. These results are consistent with Mechergui et al. (2021), who used MaxEnt to predict the habitat suitability of tree species in Tunisia, highlighting that by 2070, habitat suitability will increase from 3% to 10% and tend to shift to northern areas. These findings align with Scheiter and Higgins (2009), who predicted that by 2100, approximately 45% of African savannas will transition to woody vegetation. Additionally, decreases in grass species distribution were forecasted in Tunisia by both Soilhi et al. (2022) and Ben Mariem and Chaieb (2017). Similarly, Sarikaya and Orucu (2021) predicted a reduction in shrub species in Türkiye in a study area with climate characteristics similar to the present arid area.

### 4.3 Predicting Climate Change Effects on Vegetation Distribution and Agricultural Systems

Many studies indicate that annual temperatures are expected to rise, accompanied by a decrease in annual precipitation. This suggests that vegetation is likely to shift toward more suitable habitats at higher elevations and northern latitudes (Dibari et al. 2015; Ben Mariem and Chaieb 2017; Sarikaya and Orucu 2021; Mechergui et al. 2021; Segev et al. 2022; Almeida et al. 2023). In the temperate area shrublands and continuous grasslands are expected to expand to higher elevations (Table S2). Conversely, woods and discontinuous grasslands will find more suitable habitat conditions at lower elevations, where an increase in bare soil areas is also expected (Table S2). The expansion of bare soil and discontinuous grasslands is likely to occur due to increasingly arid conditions extending to lower elevations. This trend poses significant consequences for agricultural production in hilly and foothill areas, especially in temperate regions like temperate area. If these trends are confirmed, the promotion of shrublands over grassland ecosystems will reduce the overall palatability of rangelands, which are crucial for Mediterranean grazing systems, resulting in severe impacts on grazing system productivity (Sun et al. 2022; Durán‐Sandoval et al. 2023).

In the arid area, Random Forest, XG Boost and Neural Network showed consistency in predicting trends for the mean temperature of warmest quarter and the annual precipitation (Table S3). The shrublands are expected to shift to higher elevations (Table S3). Therefore, spontaneous vegetation will shift its distribution from current habitats to higher elevations in response to climate change and human disturbances (Dülgeroğlu and Aksoy 2019; Walther et al. 2002). In fact, flat areas are more suitable for agricultural activities such as cultivating olive plantations and cereals compared to higher areas with shallow soil depth and low fertility. On the other hand, bare soil will become more prevalent at lower altitudes (Table S3) due to wind erosion following cultivation and drier conditions (higher evaporation in the absence of vegetation) compared to the higher elevations. Additionally, lower elevations are more susceptible to erosion, resulting in less fertile and lower‐quality soil, which can affect vegetation growth. Furthermore, soil characteristics are highlighted by many researchers as crucial factors influencing plant distribution (Piri Sahragard and Ajorlo 2018).

In general, the trends of change in bioclimatic variables were consistent between the two scenarios and tended to be more pronounced in the “pessimistic” SSP 585 scenario compared to the “optimistic” SSP 245 one. In the temperate area, the annual mean temperature, annual precipitation, and precipitation seasonality showed similar trends and ranges across all four models (Tables S2 and S3). The decrease in mean annual rainfall, coupled with their expected seasonal and interannual variations, is expected to bring significant changes to grasslands, which constitute the primary forage resource for local pastoral systems (Godde et al. 2020). In Mediterranean areas, the phenological and production cycles of grasslands may shift, reducing the availability window of forage at specific elevation sites (Kavhu, Mashimbye, and Luvuno 2021; Soilhi et al. 2022; Yang et al. 2023). Moving livestock more frequently throughout the year may be an adaptive response, but it could result in higher costs for farmers of the central Apennines. Consequently, there is a risk that forage resources may remain unused, potentially leading to abandonment and negatively impacting biodiversity and associated ecosystem services (D'Ottavio et al. 2023; Francioni et al. 2019; Francioni et al. 2020; Tesei et al. 2020). Indeed, this could further lead to the abandonment of grazing areas and the encroachment of shrublands, as suggested by Filippa et al. (2019) which are expected to expand into higher elevations.

In the arid area, the increase in annual mean temperature coupled with the decrease in annual precipitation causes a shift in the distribution of woodlands, as well as bare soils and saline areas. At a local scale, the distribution of plants is consistently influenced by precipitation and temperature, which are the primary factors controlling plant productivity and composition (Ben Mariem and Chaieb 2017). Shrublands and discontinuous grasslands in arid and semi‐arid ecosystems are closely linked to variations in climatic conditions and topographic factors, and they have sharply declined under human impacts (Tlili et al. 2021).

## 5 Conclusions

Three classes of spectral vegetation indices were modeled under optimistic and pessimistic climate change scenarios in two contrasting Mediterranean study areas: Monti Sibillini (temperate area), and Sidi Makhlouf (arid area). Four machine learning algorithms have been used to model vegetation habitat suitability.

The study confirmed a shift in vegetation toward higher altitudes for shrublands and continuous grasslands in both study areas. In the temperate area, woodlands and shrublands were almost stable in terms of suitable area under the tested climate conditions, whereas discontinuous grasslands increased. In the arid area, suitable habitat for woodlands increased, while shrublands remained almost stable under both climatic scenarios, but bare soil decreased mostly under the pessimistic scenario.

Among the four machine learning algorithms, MaxEnt provided further evidence of its effectiveness in predicting habitat suitability using small presence‐only dataset and uniform climatic variables. In the temperate area, MaxEnt performed at the same level as the other algorithms, moreover in the arid area, it was the best performer and the only one able to predict the High‐SAVI class. However, some limitations in this study could be represented by the spatial resolution employed and the Worldclim data itself due to their autocorrelation.

This study contributes to a finer‐scale assessment of the impacts of climate change on the future distribution of vegetation. Similar methodologies could help inform stakeholders about the future impacts of climate change on agricultural systems, especially in developing countries with relevant socio‐economic and political constraints.

Future studies could apply the proposed methodology to address vegetation changes at the community level in several different areas across the Mediterranean basin and around the world, where significant vegetation changes are expected to be linked to climate change. Other studies could evaluate the ability of MaxEnt to improve the performance of predictive models in ensemble learning with other algorithms.

## Author Contributions

**Marco Bianchini:** data curation (equal), writing – original draft (equal). **Mohamed Tarhouni:** conceptualization (equal), methodology (equal), software (equal), validation (equal), writing – review and editing (equal). **Matteo Francioni:** conceptualization (equal), visualization (equal), writing – review and editing (equal). **Marco Fiorentini:** methodology (equal), software (equal), validation (equal). **Chiara Rivosecchi:** writing – review and editing (equal). **Jamila Msadek:** writing – review and editing (equal). **Abderrazak Tlili:** methodology (equal), writing – review and editing (equal). **Farah Chouikhi:** software (equal), validation (equal). **Marina Allegrezza:** investigation (equal), writing – review and editing (equal). **Giulio Tesei:** investigation (equal), writing – review and editing (equal). **Paola Antonia Deligios:** writing – review and editing (equal). **Roberto Orsini:** writing – review and editing (equal). **Luigi Ledda:** writing – review and editing (equal). **Maria Karatassiou:** writing – review and editing (equal). **Athanasios Ragkos:** funding acquisition (equal), project administration (equal), resources (equal). **Paride D'Ottavio:** conceptualization (equal), funding acquisition (equal), project administration (equal), resources (equal), supervision (equal), writing – review and editing (equal).

## Conflicts of Interest

The authors declare no conflicts of interest.

## Supporting information


Appendix S1.

Data S1.

## Data Availability

The data and materials underlying this article are available in the article and in its Supporting Information.
