# Supplementary material for: Modeling Climate‐Driven Vegetation Changes Under Contrasting Temperate and Arid Conditions in the Mediterranean Basin
Source: Ecol Evol. 2025 Jan 11;15(1):e70753. doi: 10.1002/ece3.70753 (PMC11724208; doi:10.1002/ece3.70753)
Supplement: Supplementary file 1 — Appendix S1. [file ECE3-15-e70753-s001.docx]

**Modelling climate-driven vegetation changes under contrasting temperate and arid conditions in the Mediterranean basin**

Marco Bianchini^1^, Mohamed Tarhouni^2*^, Matteo Francioni^1*^, Marco Fiorentini^1^, Chiara Rivosecchi^1,3^, Jamila Msadek^2^, Abderrazak Tlili^2^, Farah Chouikhi^2^, Marina Allegrezza^1^, Giulio Tesei^1^, Paola Antonia Deligios^1^, Roberto Orsini^1^, Luigi Ledda^1^, Maria Karatassiou^4^, Athanasios Ragkos^5^, Paride D’Ottavio^1^

^1^ Department of Agricultural, Food and Environmental Sciences, Università Politecnica delle Marche, Via Brecce Bianche 10, 60131 Ancona, Italy

^2^ Pastoral Ecosystems, Spontaneous Plants and Associated Microorganisms Laboratory, Arid Regions Institute-University of Gabes, Route of Djerba km 22.5, 4100 Medenine, Tunisia

^3^ Department of Civil, Constructional and Environmental Engineering, Sapienza University of Rome, Via Eudossiana 18, 00184 Rome, Italy

^4^ Laboratory of Rangeland Ecology, School of Forestry and Natural Environment, Aristotle University of Thessaloniki, P.O. Box 286, 54124 Thessaloniki, Greece

^5^ Agricultural Economics Research Institute, Hellenic Agricultural Organization - DIMITRA, Kourtidou 56-58, 11528 Athens, Greece

*Corresponding authors:

*Mohamed Tarhouni*

Pastoral Ecosystems, Spontaneous Plants and Associated Microorganisms Laboratory, Arid Regions Institute-University of Gabes, 4100 Medenine, Tunisia – [medhtarhouni@yahoo.fr](mailto:medhtarhouni@yahoo.fr)

*Matteo Francioni*

Department of Agricultural, Food and Environmental Sciences, Università Politecnica delle Marche, Via Brecce Bianche 10, 60131 Ancona, Italy – [m.francioni@staff.univpm.it](mailto:m.francioni@staff.univpm.it)

**Appendix**

**Table S1.** Pearson’s correlation test matrix for Monti Sibillini (top) and Sidi Makhlouf (bottom) case studies. Bold numbers indicate Pearson’s r values ≥ 0.75 and ≤ -0.75, which (Mechergui et al., 2021)used to identify discarded variables. Shaded columns show the retained variables: in green the selected ones, in yellowish the recovered from the discarded ones according to Wegmann et al. (2016).

**Table S1 (continue).** Pearson’s correlation test matrix for Monti Sibillini (top) and Sidi Makhlouf (bottom) case studies. Bold numbers indicate Pearson’s r values ≥ 0.75 and ≤ -0.75, which Mechergui et al. (2021) used to identify discarded variables. Shaded columns show the retained variables: in green the selected ones, in yellowish the recovered from the discarded ones according to Wegmann et al. (2016).

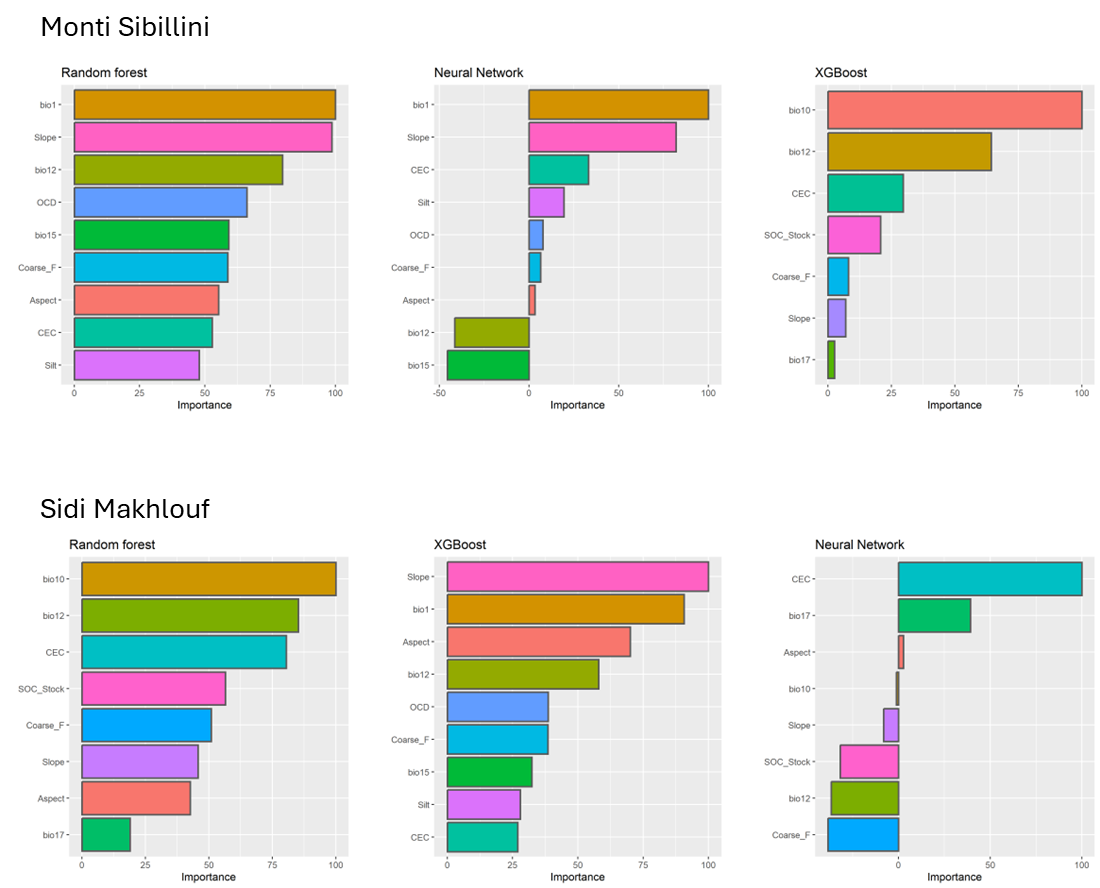


**Figure S1.** Variable importance charts for Random Forest, XG Boost, and Neural Network algorithms for the two study areas.

| 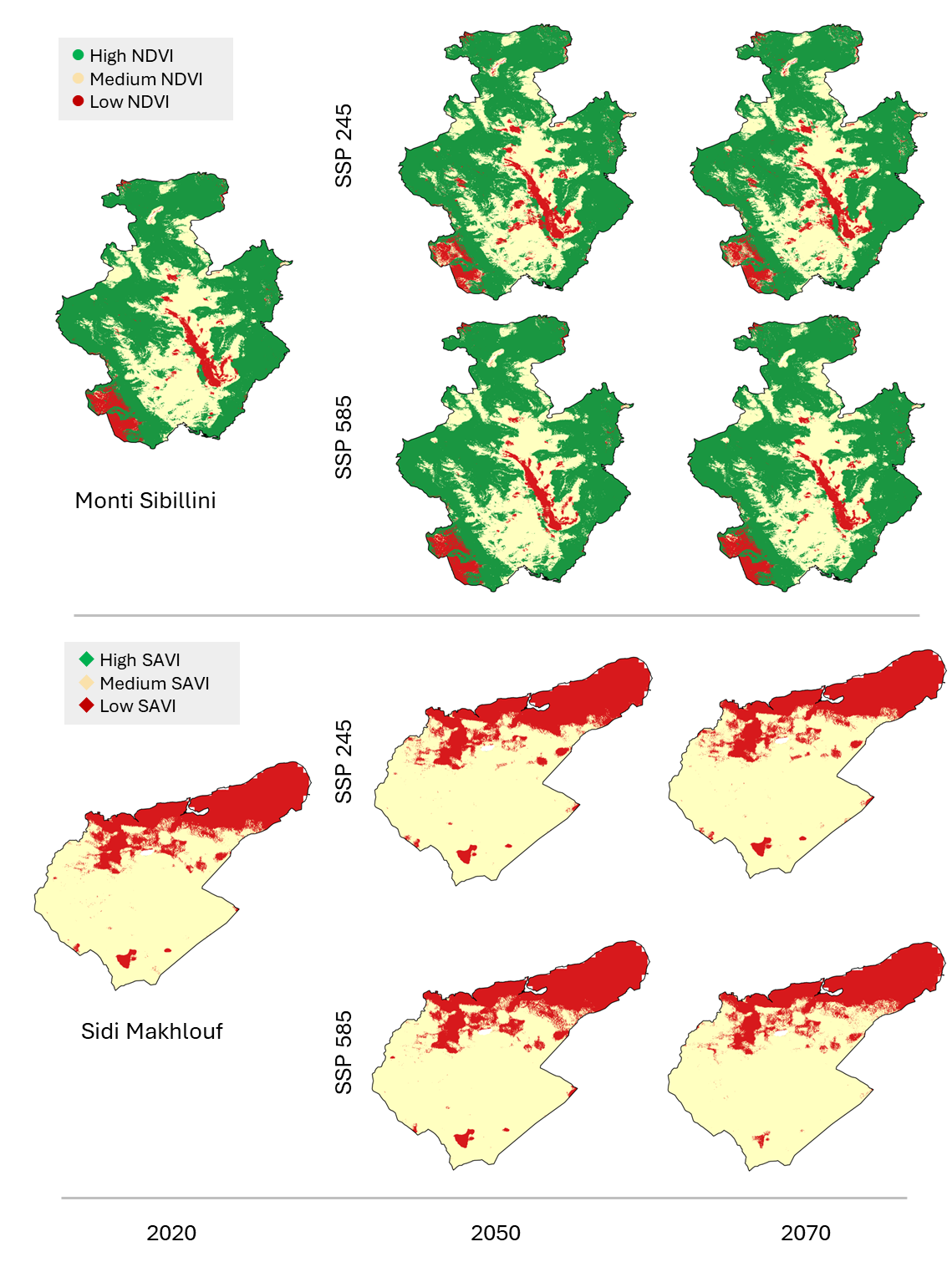  **Figure S2.** Vegetation changes predicted by Random Forest for the two study areas across three periods (2020, 2050, and 2070) and two climatic scenarios (SSP 245 and SSP 585).  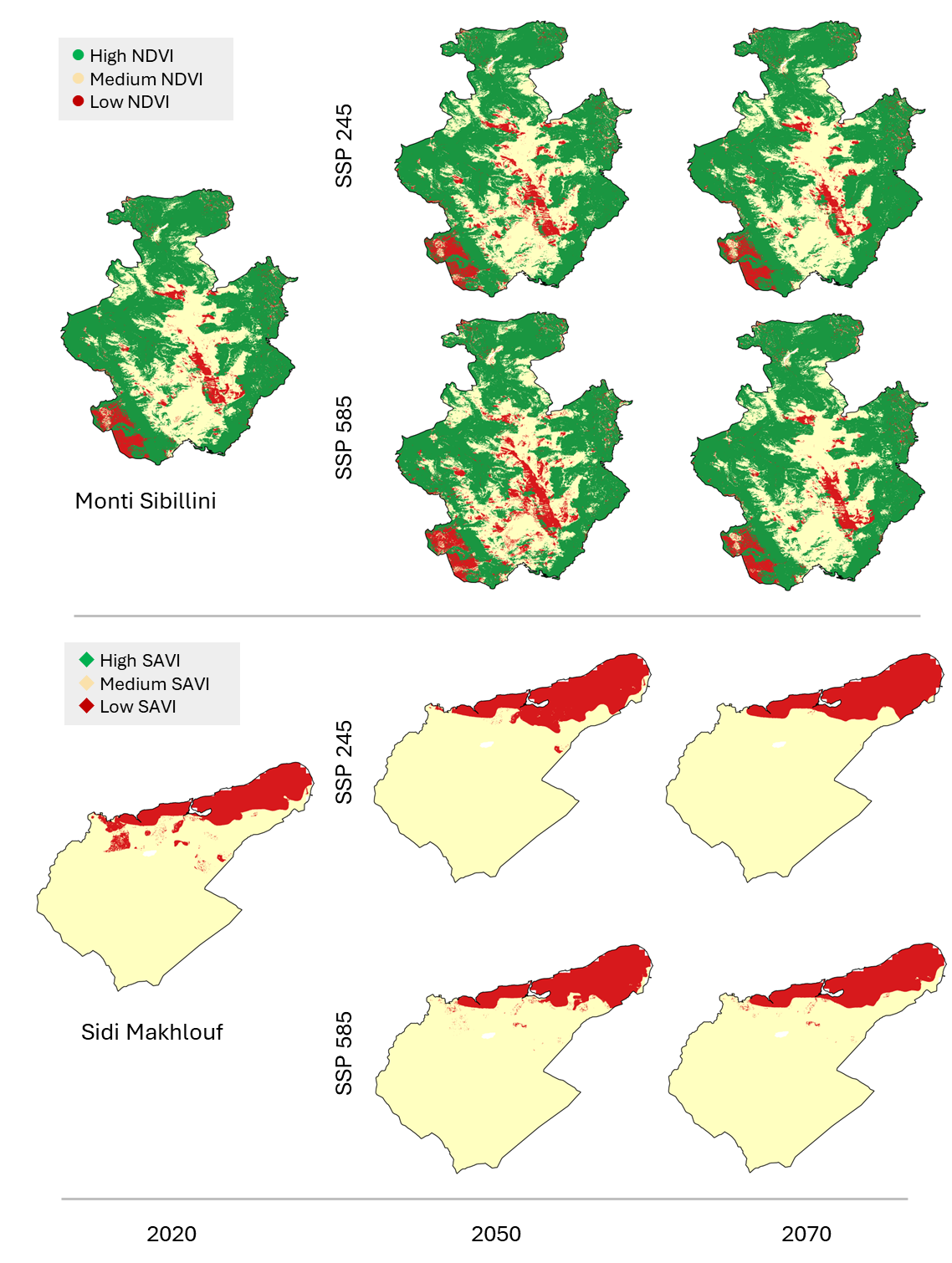  **Figure S3.** Vegetation changes predicted by XG Boost for the two study areas across three periods (2020, 2050, and 2070) and two climatic scenarios (SSP 245 and SSP 585).  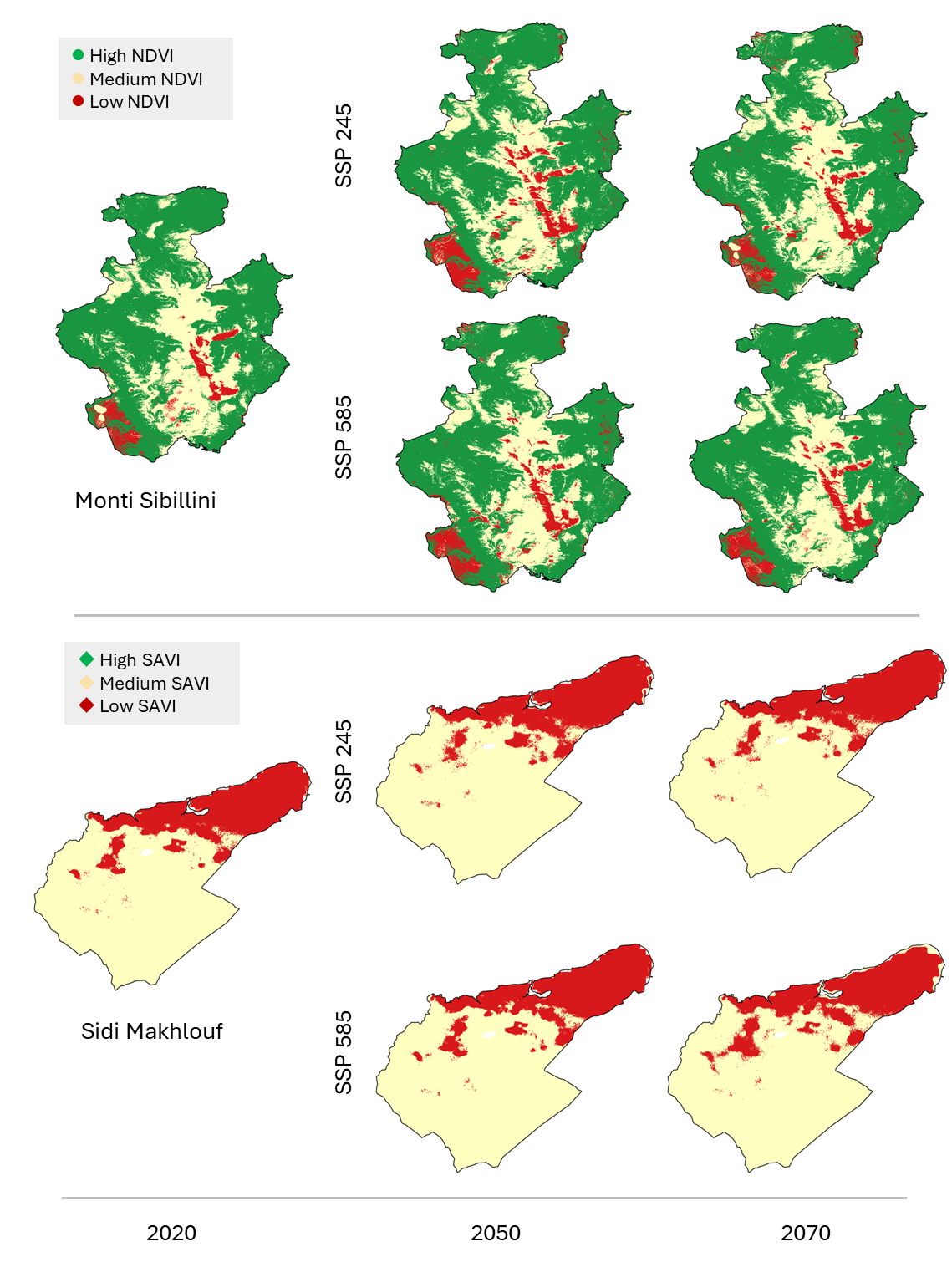  **Figure S4.** Vegetation changes predicted by Neural Network for the two study areas across three periods (2020, 2050, and 2070) and two climatic scenarios (SSP 245 and SSP 585). |
| --- |

**Table S2.** Environmental variable changes in the two climatic scenarios (SSP 245 and SSP 585) for the three Vegetation Index classes in Monti Sibillini.

|  |  |  | **MaxEnt** | | | | **Neural Network** | | | | **Random Forest** | | | | **XG Boost** | | | |
| --- | --- | --- | --- | --- | --- | --- | --- | --- | --- | --- | --- | --- | --- | --- | --- | --- | --- | --- |
| **NDVI**  **Class** | **Variable** | **Current** | **SSP 245** | | **SSP 585** | | **SSP 245** | | **SSP 585** | | **SSP 245** | | **SSP 585** | | **SSP 245** | | **SSP 585** | |
|  |  | **2020** | **2050** | **2070** | **2050** | **2070** | **2050** | **2070** | **2050** | **2070** | **2050** | **2070** | **2050** | **2070** | **2050** | **2070** | **2050** | **2070** |
| **High** | Area (ha) | 35,103 | -289 | 29 | -418 | 295 | -1,993 | -1,391 | -1,576 | 129 | -405 | -332 | 111 | -149 | -803 | 195 | -2,014 | 207 |
|  | Elevation (m a.s.l.) | 1,040 | -26 | -42 | -39 | -44 | -17 | -18 | -16 | -9 | -24 | -23 | -30 | -29 | -24 | -24 | -24 | -25 |
|  | Slope (°) | 23 | 2 | 2 | 2 | 1 | 0 | 0 | 0 | 0 | 0 | 0 | 0 | 0 | 0 | 0 | 0 | 0 |
|  | Aspect (°) | 180 | -3 | -3 | -1 | 6 | -5 | -2 | -1 | 0 | 2 | 2 | 2 | 2 | 0 | 3 | 1 | 2 |
|  | bio1 (°C) | 9 | 3 | 4 | 3 | 5 | 3 | 4 | 3 | 5 | 3 | 4 | 3 | 5 | 3 | 4 | 3 | 5 |
|  | bio12 (mm) | 893 | -57 | -49 | -94 | -45 | -58 | -52 | -95 | -44 | -59 | -51 | -95 | -46 | -59 | -51 | -94 | -45 |
|  | bio15 (%) | 21 | 1 | -4 | -1 | 2 | 1 | -4 | -1 | 2 | 1 | -4 | -1 | 2 | 1 | -4 | -1 | 2 |
| **Medium** | Area (ha) | 22,735 | -1,185 | -951 | 918 | -497 | -797 | 91 | -533 | -1,389 | -281 | -412 | 25 | 200 | -70 | 6 | -571 | -137 |
|  | Elevation (m a.s.l.) | 1,295 | 64 | 85 | 76 | 72 | 176 | 183 | 188 | 188 | 179 | 179 | 213 | 205 | 176 | 203 | 152 | 198 |
|  | Slope (°) | 19 | -3 | -3 | -2 | -2 | -2 | -2 | -2 | -2 | -1 | -1 | -1 | -1 | -2 | -2 | -1 | -2 |
|  | Aspect (°) | 192 | -12 | -12 | -10 | -27 | 12 | 4 | 0 | -1 | -8 | -7 | -8 | -6 | -14 | -10 | -19 | -8 |
|  | bio1 (°C) | 8 | 2 | 3 | 3 | 4 | 2 | 3 | 2 | 3 | 2 | 3 | 2 | 3 | 2 | 3 | 2 | 3 |
|  | bio12 (mm) | 916 | -50 | -45 | -88 | -37 | -39 | -28 | -72 | -23 | -40 | -33 | -73 | -22 | -40 | -30 | -79 | -23 |
|  | bio15 (%) | 21 | 1 | -4 | -1 | 2 | 1 | -4 | -1 | 2 | 1 | -4 | -1 | 2 | 1 | -4 | -1 | 2 |
| **Low** | Area (ha) | 12,271 | 1,474 | 922 | -500 | 201 | 2,790 | 1,300 | 2,110 | 1,260 | 686 | 745 | -136 | -50 | 872 | -200 | 2,585 | -70 |
|  | Elevation (m a.s.l.) | 1,335 | -23 | -9 | -21 | 30 | -55 | -73 | -92 | -39 | -5 | -6 | -82 | -72 | -60 | -124 | -20 | -86 |
|  | Slope (°) | 19 | -1 | 0 | -1 | 0 | -1 | -3 | -3 | -2 | -2 | -2 | -3 | -3 | -2 | -3 | -2 | -3 |
|  | Aspect (°) | 190 | 11 | 12 | 12 | 10 | 8 | 15 | 17 | 19 | 26 | 22 | 35 | 29 | 20 | 19 | 20 | 21 |
|  | bio1 (°C) | 8 | 3 | 4 | 3 | 4 | 3 | 4 | 3 | 5 | 2 | 4 | 3 | 5 | 3 | 4 | 3 | 5 |
|  | bio12 (mm) | 914 | -62 | -50 | -97 | -41 | -69 | -62 | -110 | -51 | -61 | -52 | -107 | -57 | -65 | -64 | -98 | -56 |
|  | bio15 (%) | 22 | 1 | -4 | -1 | 2 | 1 | -4 | -1 | 2 | 1 | -4 | -1 | 2 | 1 | -4 | -1 | 2 |

bio1 = Annual Mean Temperature; bio12 = Annual Precipitation; bio 15 = Precipitation Seasonality (Coefficient of Variation)

**Table S3.** Environmental variable changes in the two climatic scenarios (SSP 245 and SSP 585) for the three Vegetation Index classes in Sidi Makhlouf.

|  |  |  | **MaxEnt** | | | | **Neural Network** | | | | **Random Forest** | | | | **XG Boost** | | | |
| --- | --- | --- | --- | --- | --- | --- | --- | --- | --- | --- | --- | --- | --- | --- | --- | --- | --- | --- |
| **SAVI**  **Class** | **Variable** | **Current** | **SSP 245** | | **SSP 585** | | **SSP 245** | | **SSP 585** | | **SSP 245** | | **SSP 585** | | **SSP 245** | | **SSP 585** | |
|  |  | **2020** | **2050** | **2070** | **2050** | **2070** | **2050** | **2070** | **2050** | **2070** | **2050** | **2070** | **2050** | **2070** | **2050** | **2070** | **2050** | **2070** |
| **High** | Area (ha) | 8,629 | 4,221 | 2,119 | 6,711 | 7,241 | . | . | . | . | . | . | . | . | . | . | . | . |
|  | Elevation (m a.s.l.) | 36 | -6 | -6 | -8 | -10 | . | . | . | . | . | . | . | . | . | . | . | . |
|  | Slope (°) | 5 | 0 | -1 | -1 | -1 | . | . | . | . | . | . | . | . | . | . | . | . |
|  | Aspect (°) | 178 | 29 | 39 | 2 | 14 | . | . | . | . | . | . | . | . | . | . | . | . |
|  | bio1 (°C) | 29 | 2 | 3 | 3 | 4 | . | . | . | . | . | . | . | . | . | . | . | . |
|  | bio12 (mm) | 197 | -2 | 3 | -13 | -4 | . | . | . | . | . | . | . | . | . | . | . | . |
|  | bio15 (%) | 2 | 0 | 0 | 0 | 0 | . | . | . | . | . | . | . | . | . | . | . | . |
| **Medium** | Area (ha) | 32,684 | 642 | 948 | -416 | 1,527 | -40 | -46 | 323 | 713 | 26 | 358 | -486 | 1,699 | -1,650 | -1,009 | 326 | 800 |
|  | Elevation (m a.s.l.) | 45 | 14 | 16 | 18 | 16 | 3 | 3 | 3 | 2 | 4 | 3 | 4 | 3 | 0 | 0 | -1 | -1 |
|  | Slope (°) | 6 | 1 | 1 | 1 | 1 | 0 | 0 | 0 | 0 | 0 | 0 | 0 | 0 | 0 | 0 | 0 | 0 |
|  | Aspect (°) | 176 | 6 | -3 | -2 | 2 | 2 | 3 | 1 | 4 | 4 | 4 | 4 | 4 | 0 |  | 0 |  |
|  | bio1 (°C) | 29 | 3 | 4 | 3 | 5 | 2 | 4 | 3 | 5 | 2 | 4 | 3 | 5 | 2 | 3 | 3 | 5 |
|  | bio12 (mm) | 196 | -4 | 0 | -15 | -7 | -4 | 1 | -15 | -7 | -4 | 1 | -15 | -7 | -4 | 1 | -14 | -6 |
|  | bio15 (%) | 2 | 0 | 0 | 0 | 0 | 0 | 0 | 0 | 0 | 0 | 0 | 0 | 0 | 0 | 0 | 0 | 0 |
| **Low** | Area (ha) | 24,867 | -4,864 | -3,066 | -6,295 | -8,768 | 40 | 46 | -402 | -713 | -26 | -358 | 486 | -1,699 | 1,650 | 1,009 | -326 | -800 |
|  | Elevation (m a.s.l.) | 32 | -11 | -12 | -12 | -12 | -15 | -15 | -15 | -14 | -14 | -14 | -14 | -16 | -17 | -17 | -18 | -18 |
|  | Slope (°) | 5 | -1 | -1 | -1 | -1 | -1 | -1 | -1 | -1 | -1 | -1 | -1 | -1 | -1 | -2 | -2 | -2 |
|  | Aspect (°) | 176 | -29 | -22 | -1 | -20 | -4 | -5 | -2 | -7 | -10 | -12 | -11 | -12 | 2 | 4 | 2 | 4 |
|  | bio1 (°C) | 29 | 2 | 3 | 3 | 4 | 2 | 3 | 2 | 4 | 2 | 3 | 3 | 4 | 2 | 4 | 2 | 4 |
|  | bio12 (mm) | 199 | 1 | 5 | -11 | -3 | 3 | 9 | -8 | -1 | 2 | 8 | -9 | -1 | 7 | 12 | -4 | 5 |
|  | bio15 (%) | 2 | 0 | 0 | 0 | 0 | 0 | 0 | 0 | 0 | 0 | 0 | 0 | 0 | 0 | 0 | 0 | 0 |

bio1 = Annual Mean Temperature; bio12 = Annual Precipitation; bio 15 = Precipitation Seasonality (Coefficient of Variation)

**References**

Mechergui, K., Altamimi, A. S., Jaouadi, W., Naghmouchi, S., & El Wellani, S. (2021). Modelling current and future potential distributions of *Vachellia tortilis* (Forssk.) Hayne subsp. *raddiana* (Savi.) Brenan var. *raddiana* under climate change in Tunisia. *African Journal of Ecology*, *59*(4), 944–958. https://doi.org/10.1111/aje.12892

Wegmann, M., Leutner, B., & and Dech, S. (2016). *Remote Sensing and GIS for Ecologist: Using Open Source Software*. Pelagic Publishing, UK.
